# Recannulation of the right axillary artery in reoperative proximal thoracic aortic surgery is safe

**DOI:** 10.1093/icvts/ivac020

**Published:** 2022-02-07

**Authors:** Paul-Cătălin Puiu, Clarence Pingpoh, Maximilian Kreibich, Martin Czerny, Emmanuel Zimmer, Friedhelm Beyersdorf, Matthias Siepe

**Affiliations:** 1 Department of Cardiovascular Surgery, University Heart Center Freiburg—Bad Krozingen, Bad Krozingen, Germany; 2 Medical Faculty, Albert-Ludwigs-University Freiburg, Freiburg, Germany

**Keywords:** Aortic surgery, Axillary artery cannulation, Axillary artery, Reoperative aortic surgery, Recannulation, Cannulation technique

## Abstract

**OBJECTIVES:**

To evaluate the safety and efficacy of recannulating the axillary artery in reoperative proximal thoracic aortic surgery.

**METHODS:**

Between 2008 and 2020, we evaluated patients who underwent reoperative proximal thoracic aortic surgery. The patients were divided into 2 groups: (i) patients with no previous right axillary artery cannulation (primary cannulation group) and (ii) patients with a previous cannulated right axillary artery (recannulation group). We analysed the intraoperative data, cannulation-related complications, postoperative outcomes and compared the 2 groups (primary cannulation versus recannulation).

**RESULTS:**

The patient (*n* = 132) baseline characteristics did not differ significantly between the 2 groups. There was no statistically significant difference in regard to the duration of surgery, cardiopulmonary bypass, aortic cross-clamp and antegrade cerebral perfusion time nor in regard to the total number of patients with cannulation-related complications between the 2 groups [*n* = 8 (8.0%) vs *n* = 1 (3.1%), *P* = 0.34]. The incidence of iatrogenic axillary artery dissection, iatrogenic aortic dissection, iatrogenic aortic dissection leading to death, axillary artery thrombosis, need for surgical repair, brachial plexus injury rates, malperfusion, high perfusion resistance and hyperperfusion syndrome revealed no significant difference between the 2 groups (*P* > 0.05). There were 11 (11.0%) cases of stroke in the primary cannulation group and 1 (3.1%) in the recannulation group (*P* = 0.18).

**CONCLUSIONS:**

Recannulation of the right axillary artery in reoperative proximal thoracic aortic surgery is not associated with worse clinical outcomes and can be safely and effectively performed, also representing a preventive and rescue measure. Previous cannulation of the axillary artery should not hinder the decision for recannulation.

**Figure:**
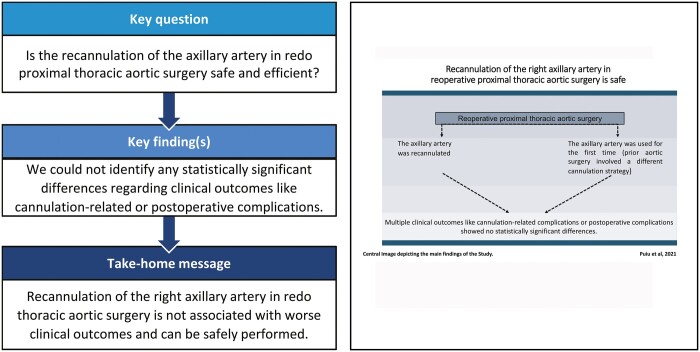

## INTRODUCTION

Cannulation of the right axillary artery (RAA) has emerged as a routine method in aortic surgery for establishing selective antegrade cerebral perfusion (ACP) [1–4]. With increasing liberal use of axillary artery cannulation and increasing number of cardiac reoperations, the number of patients with previously cannulated RAA also increases. It has been previously shown that reoperative surgery *per se* is not a major risk factor for poor outcomes, provided that strategies for preventing intraoperative adverse events are being used [5].

The aim of this study was to evaluate the safety and efficacy of recannulating the axillary artery in redo proximal thoracic aortic surgery.

## PATIENTS AND METHODS

### Study population and objectives

From January 2008 until January 2020, we retrospectively evaluated all patients who underwent reoperative proximal thoracic aortic surgery at the University Heart Center Freiburg—Bad Krozingen, Germany. First, the entire reoperative surgery cohort was analysed and then the patients were divided into 2 groups: (i) patients with no previous RAA cannulation (primary cannulation group) and (ii) patients with a previous cannulated RAA (recannulation group). Patients with missing data were excluded from the analysis.

The retrospective study was approved by the review committee of the University of Freiburg (Germany) (Nr. 341/20). Formal consent was not required since this was a retrospective study but patient data were pseudonymized.

Patient data were reviewed and extracted from our institutional medical records and included preoperative, operative and postoperative variables. Cannulation-related complications were evaluated and included iatrogenic axillary artery dissection, iatrogenic aortic dissection, iatrogenic aortic dissection leading to death, axillary artery thrombosis, brachial plexus injury, need for surgical repair, high perfusion resistance, malperfusion and hyperperfusion syndrome.

Clinical and operative variables were defined according to the Society of Thoracic Surgeons criteria [6, 7]. Stroke was defined as a new postoperative physician-diagnosed neurological deficit confirmed by imaging methods—computed tomography or magnetic resonance imaging—and persisting >24 h. The diameter of the axillary artery was measured retrospectively perpendicular to the centreline of the vessel between the first rib and clavicle using computed tomography angiography.

In the present retrospective study, our primary outcome included the above described cannulation-related complications. Our secondary outcome comprised postoperative complications.

### Surgical technique

In our centre, we routinely cannulate the axillary artery through a vascular prosthetic graft (usually an 8-mm Dacron graft) [8] with the following exclusion criteria: aberrant subclavian artery (lusorian artery), emergency surgery with resuscitation and high perfusion pressure. After decannulation, we ligate the side-graft above the level of the native vessel with 2 non-resorbable sutures and add a double layer running 4.0 prolene suture. Our routine is the infraclavicular approach. During reoperations with recannulation of the RAA, we begin the dissection along the previous side-graft stump mobilizing the artery proximal and distal to it before recannulating it through a new side-graft anastomosed distally to the old stump. We adapt the cannula’s size to the calculated perfusion volume according to body surface area (e.g. a 20-French Cannula or a 22-French Cannula) and the tip of the cannula is placed only into the side-graft. For complete arch repair, a selective perfusion cannula is always inserted into the left carotid artery directly. For hemiarch repair, left carotid artery cannulation is only done when near-infrared spectroscopy decline or whenever the surgeon expected longer than a 30-min ACP time duration.

The indications for reoperative thoracic aorta surgery with axillary artery recannulation were: extension of the dissection, aortic aneurysmal disease with early or late progression of diameter, pseudoaneurysms, infection and to initiate cardiopulmonary bypass (CPB) before the sternotomy.

### Statistical analysis

To test the normality of data, we used the Shapiro–Wilk test. Not normally distributed continuous variables are presented as the median and interquartile range (IQR) and were compared using the Wilcoxon–Mann–Whitney test, whereas normally distributed continuous variables are presented as mean (standard deviation) and were compared using the Student’s *t*-test. Categorical and binary variables are presented as frequencies (*n*) and percentages (%) and were compared using the Pearson’s χ^2^ test, applying Fisher’s exact test when *n* < 5. Because the baseline and operative data of the cohorts did not differ significantly and because of the limited number of cases, we refrain from using a propensity score matching technique. Statistical analysis was performed using R version 3.6.1 for macOS (The R Foundation for Statistical Computing, Austria) and Stata statistical software for macOS (Stata/MP version 13.0; StataCorp, TX, USA) and with a level of significance set at *P* < 0.05.

## RESULTS

### Baseline characteristics

In the case of 32 patients, the axillary artery was recannulated, while in the primary cannulation group of 100 patients, the axillary artery was used for the first time and prior aortic surgery involved a different cannulation strategy. The patient baseline characteristics are depicted in Table 1. The median age of the study population was 65.0 years (54.0–73.0, IQR), in the primary cannulation group 65.0 years (55.5–71.0, IQR) and in the recannulation group 66.0 (47.0–75.5, IQR) with 34 (34.9%) male patients in the first group and 9 (28.1%) in the second group. Sixteen (16.0%) patients had Marfan’s syndrome in the primary cannulation group and 3 (9.4%) in the recannulation group; 6 (6.0%) had a bicuspid aortic valve in the former group and 3 (9.4%) in the latter.

**Table 1:** Patient baseline characteristics

Variable	All patients (*n* = 132)	Primary cannulation (*n* = 100)	Recannulation group (*n* = 32)	*P*-value
Age (years)	65.0 (54.0–73.0)	65.0 (55.5–71.0)	66.0 (47.0–75.5)	0.99
Male	43 (32.6)	34 (34.0)	9 (28.1)	0.54
BMI (kg/m^2^)	26.0 (24.0–28.3)	25.6 (24.0–28.1)	27.0 (25.5–28.8)	0.11
Arterial hypertension	107 (81.1)	86 (86.0)	21 (65.6)	0.010
Pulmonary hypertension	5 (3.8)	3 (3.0)	2 (6.2)	0.40
Diabetes mellitus	13 (9.8)	10 (10.0)	3 (9.4)	0.92
Dyslipidaemia	47 (35.6)	38 (38.0)	9 (28.1)	0.31
Smoking history	50 (37.9)	42 (42.0)	8 (25.0)	0.084
COPD	12 (9.1)	10 (10.0)	2 (6.2)	0.52
CKD	25 (18.9)	20 (20.0)	5 (15.6)	0.58
PAOD	2 (1.5)	2 (2.0)	0 (0.0)	0.42
Previous stroke	25 (18.9)	18 (18.0)	7 (21.9)	0.63
Carotid disease	5 (3.8)	5 (5.0)	0 (0.0)	0.20
Left ventricular EF				
≤20%	0 (0)	0 (0)	0 (0)	1.00
21–30%	6 (4.5)	5 (5.0)	1 (3.1)	
31–50%	8 (6.1)	6 (6.0)	2 (6.2)	
>50%	118 (89.4)	89 (89.0)	29 (90.6)	
Coronary artery occlusive disease	16 (12.1)	12 (12.0)	4 (12.5)	0.94
Previous myocardial infarction	3 (2.3)	2 (2.0)	1 (3.1)	0.71
Moderate to severe mitral disease	9 (6.8)	8 (8.0)	1 (3.1)	0.34
Moderate to severe aortic disease	30 (22.7)	21 (21.0)	9 (28.1)	0.40
Marfan’s syndrome	19 (14.4)	16 (16.0)	3 (9.4)	0.35
Bicuspid aortic valve	9 (6.8)	6 (6.0)	3 (9.4)	0.51
Pericardial effusion	1 (0.8)	1 (1.0)	0 (0.0)	0.57
Shock	1 (0.8)	1 (1.0)	0 (0.0)	0.57
Aneurysm without dissection	48 (36.4)	37 (37.0)	11 (34.4)	0.79
Aortic dissection				0.61
A	65 (49.2)	46 (46.0)	19 (59.4)	
B	9 (6.8)	8 (8.0)	1 (3.1)	
Non A-non B	5 (3.8)	4 (4.0)	1 (3.1)	
Porcelain aorta	4 (3.0)	3 (3.0)	1 (3.1)	0.97
Preoperative haemoglobin	13.7 (12.4–15.0)	13.0 (11.0–14.9)	13.9 (12.9–15.0)	0.17
Right axillary artery diameter (mm)	8.2 (7.5–9.0)	8.1 (7.5–9.0)	8.5 (7.8–9.0)	0.43

Data are presented as median (interquartile range) for continuous variables and as *n* (%) for binary and categorical variables.

BMI: body mass index; CKD: chronic kidney disease; COPD: chronic obstructive pulmonary disease; EF: ejection fraction; PAOD: peripheral artery occlusive disease.

### Operative data

Intraoperative data are summarized in Table 2 including the performed surgeries. The duration of surgery [196.5 min (161.5–238, IQR) vs 195.5 min (167.5–263, IQR) *P* = 0.51], CPB time [113.5 min (82.5–139.5, IQR) vs 118 min (59.5–173, IQR), *P* = 0.93], aortic cross-clamp time [60 min (37–88, IQR) vs 47 min (37–89, IQR), *P* = 0.86] and antegrade cerebral perfusion time [24.85 min (24–27.85, IQR) vs 24.7 min (23.95–25.75, IQR) *P* = 0.47] showed no statistically significant difference between the 2 groups, primary and recannulation, respectively. The cannulation of the artery was performed by using a side-graft in 69 (69.0%) patients in the primary cannulation group and in 30 (93.8%) in the recannulation group, *P* = 0.005.

**Table 2:** Operative data

Variable	All patients (*n* = 132)	Primary cannulation (*n* = 100)	Recannulation group (*n* = 32)	*P*-value
Emergency	13 (9.8)	12 (12.0)	1 (3.1)	0.14
Type of surgery				
Ascending aortic replacement	102 (77.3)	79 (79.0)	23 (71.9)	0.40
Arch replacement	104 (78.8)	80 (80.0)	24 (75.0)	0.55
Ascending and arch replacement	48 (36.4)	41 (41.0)	7 (21.9)	0.050
Ascending and root replacement	14 (10.6)	10 (10.0)	4 (12.5)	0.69
Root, ascending and arch replacement	26 (19.7)	17 (17.0)	9 (28.1)	0.17
Side-graft cannulation	99 (75.0)	69 (69.0)	30 (93.8)	0.005
Concomitant operation	13 (9.8)	10 (10.0)	3 (9.4)	0.92
Aortic valve resuspension	37 (28.0)	26 (26.0)	11 (34.4)	0.36
Aortic valve replacement	3 (2.3)	2 (2.0)	1 (3.1)	0.71
Aortic valve repair	13 (9.8)	9 (9.0)	4 (12.5)	0.56
CABG	6 (4.5)	6 (6.0)	0 (0.0)	0.16
Mitral surgery	9 (6.8)	9 (9.0)	0 (0.0)	0.079
Tricuspid surgery	11 (8.3)	10 (10.0)	1 (3.1)	0.22
Other	446.6 (369.6–557.15)	455.5 (376–550.65)	393 (361.5–582.5)	0.79
Duration of surgery	196 (163.5–241)	196.5 (161.5–238)	195.5 (167.5–263)	0.51
CPB time	114.5 (82–146)	113.5 (82.5–139.5)	118 (59.5–173)	0.93
ACC time	58.5 (37–88.5)	60 (37–88)	47 (37–89)	0.86
ACP	24.8 (24–27.8)	24.85 (24–27.85)	24.7 (23.95–25.75)	0.47
Lowest BT (°C)				

Data are presented as median (interquartile range) for continuous variables and as *n* (%) for binary and categorical variables. Time in min.

ACC: aortic cross-clamp; ACP: antegrade cerebral perfusion; BT: body temperature; CABG: coronary artery bypass grafting; CPB: cardiopulmonary bypass.

### Recannulation-related complications

There was no statistically significant difference in regard to the total number of patients with cannulation-related complications between the primary cannulation group and recannulation group [*n* = 8 (8.0%) vs *n* = 1 (3.1%), *P* = 0.34] (Table 3). The incidence of iatrogenic axillary artery dissection, iatrogenic aortic dissection, iatrogenic aortic dissection leading to death, axillary artery thrombosis, need for surgical repair, brachial plexus injury rates, malperfusion, high perfusion resistance and hyperperfusion syndrome all revealed no significant difference between the 2 groups (*P* > 0.05). No case of axillary cannulation attempt and break-off for technical reasons was documented or remembered by the aortic team.

**Table 3:** Cannulation-related complications

Variable	All patients (*n* = 132)	Primary cannulation (*n* = 100)	Recannulation group (*n* = 32)	*P*-value
Iatrogenic axillary artery dissection	2 (1.5)	2 (2.0)	0 (0.0)	0.42
Progression to iatrogenic aortic dissection	2 (1.5)	2 (2.0)	0 (0.0)	0.42
Iatrogenic aortic dissection leading to death	0 (0)	0 (0)	0 (0)	
Axillary artery thrombosis	0 (0)	0 (0)	0 (0)	
Brachial plexus injury	3 (2.3)	2 (2.0)	1 (3.1)	0.71
Need for surgical repair	1 (0.8)	1 (1.0)	0 (0.0)	0.57
High perfusion resistance	1 (0.8)	1 (1.0)	0 (0.0)	0.57
Malperfusion	0 (0)	0 (0)	0 (0)	
Hyperperfusion syndrome	1 (0.8)	1 (1.0)	0 (0.0)	0.57
Total patients with complications	9 (6.8)	8 (8.0)	1 (3.1)	0.34

Data are presented as *n* (%) for binary and categorical variables.

### Postoperative outcomes

The postoperative outcomes are summarized in Table 4. There were no case of intraoperative death. There were 11 (11.0%) cases of stroke in the primary cannulation group and 1 (3.1%) in the recannulation group, *P* = 0.18. Other postoperative complications also revealed no statistically significant differences. The need for transfusion of erythrocyte concentrates and platelet concentrates were numeric slightly higher in the recannulation group but did not attained statistical significance (*P* = 0.67; *P* = 0.23).

**Table 4:** Postoperative outcomes

Variable	All patients (*n* = 132)	Primary cannulation (*n* = 100)	Recannulation group (*n* = 32)	*P*-value
Erythrocyte concentrates tranfusion	4 (2–8)	4 (2–7.5)	5 (2–9)	0.67
Platelet concentrates tranfusion	1 (1–4)	1 (1–4)	2.5 (1–6)	0.23
Fresh frozen plasma transfusion	6 (4–8.5)	6 (4–9)	6 (4–8)	0.94
Stroke	12 (9.1)	11 (11.0)	1 (3.1)	0.18
Postoperative paraplegia	0 (0)	0 (0)	0 (0)	
Postoperative dialysis	9 (6.8)	7 (7.0)	2 (6.2)	0.88
Tracheostomy	6 (4.5)	6 (6.0)	0 (0.0)	0.16
Pneumonia	11 (8.3)	10 (10.0)	1 (3.1)	0.22
ECLS	6 (4.5)	5 (5.0)	1 (3.1)	0.66
Sepsis	7 (5.3)	5 (5.0)	2 (6.2)	0.78
Intensive care unit stay (days)	5.765 (2–9.35)	5.765 (2–9.35)	5.5 (2–9.5)	0.93
Hospital stay (days)	19.5 (15–27)	20 (16–28)	17.5 (14–24.5)	0.16
30-day mortality	16 (12.1)	10 (10.0)	6 (18.8)	0.19
Intraoperative death	0 (0)	0 (0)	0 (0)	

Data are presented as median (interquartile range) for continuous variables and as *n* (%) for binary and categorical variables.

ECLS: extracorporeal life support.

## DISCUSSION

In the present study, we compared clinical outcomes of recannulation of the RAA with primary cannulation of the RAA in reoperative proximal thoracic aortic surgery and found no statistically significant differences neither in regard to cannulation-related complications nor postoperative complications.

Aortic and cardiac reoperations are complex procedures with high operative risk requiring careful preoperative planning, preventive strategies and compensatory rescue measures. Safely and promptly establishing CPB is of the utmost importance, especially in the event of a re-entry catastrophic injury allowing the repair and limiting the haemodynamic instability. It has been previous shown that careful preoperative planning using computed tomography scanning and using peripheral arterial cannulation is associated with less injuries to vital structures [9].

RAA is supported by growing evidence as the arterial cannulation site of first choice in aortic surgery. It allows establishing selective ACP, avoiding flow reversal and it has been shown to be more beneficial than other cannulation sites in terms of early embolic stroke, mortality and neurological dysfunction [1, 2, 10]. The 2014 European Society of Cardiology Guidelines on the diagnosis and treatment of aortic diseases recommends that the axillary artery should be considered as first choice for cannulation for surgery of the aortic arch and in aortic dissection (class of recommendation IIa, level of evidence C) aided by the use of ACP and transcranial oxygen saturation assessment [11].

In reoperative aortic surgery, cannulation of the RAA represents both a preventive strategy and a compensatory rescue measure—selective ACP and hypothermia can be initiated during or before re-entry and surgical dissection; in the case of a catastrophic injury repair can be initiated undelayed.

Shetty *et al*. [12] reported their favourable experience with the re-use of the axillary artery as cannulation site in 2 patients undergoing repeat aortic surgery. Both patients were reoperated because of pseudoaneurysms and in both cases a new 8-mm side-graft was anastomosed and used for arterial inflow. The patients recovered uneventful. Bowers and Matur [13] also reported their experience with RAA recannulation in 7 patients. All 7 patients underwent cannulation with a new side-graft and they concluded that recannulation is simple and safe.

The results of the present study confirm and extend these previous observations. In our series, by comparing 32 recannulation cases with 100 primary cannulations, all within a reoperative proximal thoracic aortic surgery cohort, we could not identify any statistically significant differences in regard to multiple clinical outcomes like cannulation-related complications or postoperative complications. We have previously shown that cannulation of the RAA through a side-graft is superior to direct cannulation in regard to cannulation-related complications and stroke rates [8] and our strategy is to routinely use a side-graft for redo surgery. We still cannulate directly in the case of patients requiring resuscitation and in case of emergency surgery.

Our data show that recannulation of the RAA in proximal thoracic aortic surgery is efficient and safe. All the advantages of this beneficial cannulation site are preserved—no flow reversal, the possibility of establishing selective ACP, hypothermia and CPB and in the specific case of redo surgery even expanded by the role of RAA recannulation as a preventive and rescue measure.

### Limitations

This is a retrospective study with all the inherent limits and risks for confounding and bias. Our clinical database might under-report events and risk factors and we were not able to gather late clinical outcome data or data on quality of life. The operations were performed in a high-volume centre with experience in aortic surgery and the choice of cannulation site and cerebral protection strategy was surgeon’ preference. Also, in the recannulation group, a side-graft was more often used as in the primary cannulation group, fact that could also be responsible for the rate of complications observed [8, 14].

A major limitation of the study is the limited number of cases. Furthermore, our results are not adjusted for major confounders. Larger multicentric trials should further clarify the safety of the procedure.

## Conclusions

Recannulation of the RAA in reoperative proximal thoracic aortic surgery is not associated with worse clinical outcomes and can be safely and effectively performed. In complex redo proximal thoracic aortic operations, all the reported advantages of RAA cannulation are extended by the role of preventive and rescue measure that this inflow site can play; previous cannulation of the axillary artery is not an impediment and should not hinder the decision for recannulation.

**Conflict of interest:** The conflict of interest of Martin Czerny with Terumo, Medtronic and Cryolife does not have anything in common with materials used in the study or with the conclusions. 

## Data availability statement

All relevant data are within the manuscript and its supporting information files.

## Author contributions

**Paul-Cătălin Puiu:** Conceptualization; Data curation; Formal analysis; Investigation; Methodology; Project administration; Software; Supervision; Validation; Visualization; Writing—original draft. **Clarence Pingpoh:** Conceptualization; Supervision; Validation; Visualization. **Maximilian Kreibich:** Conceptualization; Data curation; Investigation; Supervision; Validation. **Martin Czerny:** Conceptualization; Investigation; Methodology; Supervision; Validation. **Emmanuel Zimmer:** Conceptualization; Data curation; Supervision; Writing—review & editing. **Friedhelm Beyersdorf:** Conceptualization; Methodology; Project administration; Visualization; Writing—review & editing. **Matthias Siepe:** Conceptualization; Methodology; Project administration; Resources; Supervision; Writing—review & editing.

## Reviewer information

Interactive CardioVascular and Thoracic Surgery thanks Mitsuru Asano and the other, anonymous reviewer(s) for their contribution to the peer review process of this article.

## Abbreviations

**ABBREVIATIONS**
ACP: Antegrade cerebral perfusion
CPB: Cardiopulmonary bypass
IQR: Interquartile range
RAA: Right axillary artery
